# Dynamic “Molecular Portraits” of Biomembranes Drawn by Their Lateral Nanoscale Inhomogeneities

**DOI:** 10.3390/ijms22126250

**Published:** 2021-06-10

**Authors:** Roman G. Efremov

**Affiliations:** 1 M.M. Shemyakin & Yu.A. Ovchinnikov Institute of Bioorganic Chemistry, Russian Academy of Sciences, Miklukho-Maklaya Street, 16/10, 117997 Moscow, Russia; efremov@nmr.ru; Tel.: +7-903-743-16-56; 2 Department of Applied Mathematics, National Research University Higher School of Economics, Myasnitskaya ul. 20, 101000 Moscow, Russia; 3 Moscow Institute of Physics and Technology (State University), 141701 Dolgoprudny, Russia

**Keywords:** computer simulations, dynamics of lipid membranes, lipid–lipid H-bonding, lateral heterogeneity of membrane, mechanisms of nanodomain formation, model biomembranes, molecular dynamics, mosaicity of membrane surface, physico-chemical properties of lipid bilayers, spontaneously formed nanodomains

## Abstract

To date, it has been reliably shown that the lipid bilayer/water interface can be thoroughly characterized by a sophisticated so-called “dynamic molecular portrait”. The latter reflects a combination of time-dependent surface distributions of various physicochemical properties, inherent in both model lipid bilayers and natural multi-component cell membranes. One of the most important features of biomembranes is their mosaicity, which is expressed in the constant presence of lateral inhomogeneities, the sizes and lifetimes of which vary in a wide range—from 1 to 10^3^ nm and from 0.1 ns to milliseconds. In addition to the relatively well-studied macroscopic domains (so-called “rafts”), the analysis of micro- and nanoclusters (or domains) that form an instantaneous picture of the distribution of structural, dynamic, hydrophobic, electrical, etc., properties at the membrane-water interface is attracting increasing interest. This is because such nanodomains (NDs) have been proven to be crucial for the proper membrane functioning in cells. Therefore, an understanding with atomistic details the phenomena associated with NDs is required. The present mini-review describes the recent results of experimental and in silico studies of spontaneously formed NDs in lipid membranes. The main attention is paid to the methods of ND detection, characterization of their spatiotemporal parameters, the elucidation of the molecular mechanisms of their formation. Biological role of NDs in cell membranes is briefly discussed. Understanding such effects creates the basis for rational design of new prospective drugs, therapeutic approaches, and artificial membrane materials with specified properties.

## 1. Introduction

Apart from the barrier function separating contents of cells or cellular compartments from the exterior, lipid bilayer of biological membranes plays a critical role in numerous biochemical processes in the living organisms. Up to 80% of the mass in cell membranes is related to proteins, sterols, carbohydrates, and other non-lipidic components, which determine specificity and a broad spectrum of biological activities of the membranes, such as molecular and ion transport, cell communication and signaling, membrane fusion, and so on [1]. At the same time, concerted functioning of such complex supramolecular ensembles should be strictly regulated to assure fast, robust, and adequate reaction of a membrane on external/internal signals and pathological threats [2,3]. Intimate molecular details of this amazing machinery of cell membranes are far from being understood, although it has become clear that membrane lipids represent a very important “piece of the puzzle”. Instead of being a passive “sea” with polar surfaces and a hydrophobic core, where different proteins and other molecules can accomplish their functions, multicomponent lipid bilayers of cell membranes represent themselves as a highly active, dynamic, fine-tuning, and self-organizing medium [4].

Currently, it has become clear that all the roles of cell membranes mentioned above are largely due to the heterogeneity of properties of their lipid “skeleton” on different spatial and temporal scales. This is a fundamental property, which therefore requires in-depth analysis. It is obvious that the heterogeneous organization of lipid membranes is due to the physical and chemical nature of their constituent molecules, primarily amphiphilic lipids, as well as their wide diversity—apart from proteins, natural membranes contain hundreds of types of lipids and other compounds. Differences in head-groups and/or acyl chains in lipid molecules lead to a large variety of intermolecular interactions and thereby to non-ideal mixing of lipids in bilayers [5,6].

Often, when we talk about the heterogeneous nature of cell membranes, we mean their “layered” arrangement—zones with radically different physical and chemical properties are layers that alternate along the normal to the lipid bilayer plane. It is this organization of biomembranes that creates a reliable barrier that protects the contents of the cell from the external environment, ensures the correct insertion, assembling and functioning of numerous membrane proteins, membrane-active peptides, and other molecules. However, no less important are the lateral distributions of the properties of these layers. It is known that they are also heterogeneous in a number of key parameters—the density of membrane components (lipids, small molecules, water, ions, etc.). The most significant among these regions (layers) are the membrane–water interfaces. The properties of these surfaces largely determine the mechanisms of recognition of cell membranes and their model mimetics by external agents, such as proteins, peptides, and their supramolecular complexes, including viruses, etc.

It should also be kept in mind that such lateral inhomogeneities change over time, including equilibrium or quasi-equilibrium states (as far as they can be discussed in a living cell at all). As is shown below, the spatiotemporal scales of inhomogeneities (domains, clusters) vary in a wide range—from 1 to 10^3^ nm and from 0.1 ns to milliseconds. What are the properties of the membrane–water interface that exhibit inhomogeneities in their lateral distributions? The most important are: (1) their structural characteristics, expressed in terms of the density of molecules and individual atoms, as well as describing the relief of the molecular surface of the lipid bilayer; (2) the surface distribution of their hydrophobic/hydrophilic and/or electrostatic properties; (3) dynamic parameters of the membrane components due to their lateral diffusion at different spatial scales—from integral macroscopic averages to the trajectories of individual molecules and their groups.

Taken together, all these types of time-dependent inhomogeneities are usually combined within the concept of “mosaic picture” of the membrane-water interface. In other words, it is a kind of “dynamic molecular portrait” (DMP) of the membrane surface, the parameters of which are unique for lipid bilayers of a certain composition that are in specific conditions (phase state, degree of hydration, the presence of ions and “external agents”, including integral and peripheral membrane proteins, peptides, and other molecules that interact with the cell membrane). Given the specifics of the organization of the membranes, both their surfaces can be fairly accurately approximated by a plane (at least on molecular scales). This allows representation of the corresponding DMPs in the form of two-dimensional (2D) maps of the lateral distribution of properties that evolve over time. In contrast to three-dimensional (3D) objects, it is much more convenient and efficient to work with 2D distributions in the form of DMP since they can be efficiently processed using numerical methods: their average characteristics and the corresponding standard deviations can be calculated; they can be subjected to digital filtering; maps of different states of one system or different systems can be compared, visualized, etc. For more information about this, see Section 5 below. Note that similar technologies for working with DMPs are also used for the analysis of non-planar biological molecular objects; in particular, globular proteins and/or their individual structural elements, for example, alpha-helices, etc. In such cases, the DMP is created by projecting the properties of the molecular surface onto the surface of a sphere (for ball-shape objects, such as globular proteins) [7] or cylinder (for rod-like objects, such as α-helix) [8], respectively. It should be noted that the term “DMP” does not mean that it is a special computational method or model developed by the author to describe the properties of the membrane surface. In the context of this review, this is just a way of presenting data that is convenient to use when describing the time-dependent set of different physicochemical properties of the membrane-water interface (see above).

Given the fact that cell membranes are literally “stuffed” with proteins and other molecules [9], the study of the effects of lateral heterogeneity is very difficult due to the small areas of the “free” lipid bilayer. Therefore, such work is usually carried out on model systems that mimic the cell membrane—lipid bilayers consisting of one or more types of lipids. Another feature of the study of DMP for biomembranes is the range of analyzed spatiotemporal characteristics. The most studied are long-living domains (clusters) of relatively large size, which exceeds 100 nm—lipid “rafts” and microdomains. At the same time, for obvious reasons, smaller inhomogeneities—the so-called “nanodomains” (ND), or nanoclusters—are much less studied because of technical limitations of modern experimental methods. The characteristic size of NDs is less than 10 nm, which corresponds just to several tightly packed lipids. In addition, the lifetime of NDs is often below a few nanoseconds. That is why such systems still lie beyond the resolution that is suitable for their direct observation in experiments. Excellent reviews [10,11,12,13] provide a comprehensive picture of the current state in this field.

There may be a feeling that due to the averaging, such ND-related phenomena are not able to significantly affect the macroscopic properties of the lipid bilayer, but this is not so at all. In particular, the role of microscopic heterogeneities in the membranes follows from the fact that the self-organization and functioning of the most important classes of membrane protein receptors, ion channels, enzymes, etc., may critically depend on the properties of the annular lipids that form one-two nearest molecular layers (e.g., [11,14,15]). Often, characteristics of the latter are very different from the “free” lipid bilayer membrane. (Although such curvature effects on the membrane surface are purely 3D in nature, they (as with other landscape-related properties) are conveniently represented as 2D maps—e.g., via projection on the membrane plane.) Another example is that local (of the order of 10 nm) curvature defects on the membrane surface practically determine the binding of a number of important membrane-active peptides in these regions [16], affect the processes of membrane fusion [17], etc. Thus, it is necessary to understand the atomistic aspects of the formation and evolution of DMPs of the cell membranes. This approach is based on a detailed analysis of NDs: their identification, characterization, and delineation of the corresponding atomistic mechanisms. Some aspects of the problem are discussed in comprehensive recent reviews [10,11,12,13,18], but here, the author would like to express his thoughts on this problem based on the results of his own long-term research in this area.

## 2. Characteristics of Lateral Heterogeneities in Lipid Bilayers

The nanoscale lateral inhomogeneities (nanodomains, NDs) in the lipid bilayer of cellular and model membranes can be divided into two large groups: (i) arising spontaneously and (ii) arising as a result of the influence of external factors (in relation to the components of the lipid bilayer) like other molecules (for example, peptides and proteins), changes in environmental parameters (temperature, degree of hydration, presence of ions, etc.), curvature of the membranes, etc. Undoubtedly, these processes are interrelated, since in order for a certain type of ND-based DMP to arise in the membrane under the influence of external factors, it is necessary that the undisturbed lipid bilayer itself (including water molecules and ions) is able in principle to spontaneously form such nanoscale structures. Here, we review only the spontaneously formed lateral NDs and their characteristics, while the externally induced inhomogeneities, which are much more involved in the biological action of the membranes, require separate consideration.

The sections below are organized as follows: First, we discuss the available data on the detection and characterization of NDs in experiments with model lipid bilayers, and also demonstrate the possibilities of observing nanosized objects in the membranes of living cells. Then, we focus on critically examining the lessons of studying atomic-scale lateral inhomogeneities in membranes with the full arsenal of available modern tools. Finally, we turn to the results of the NDs analysis using computational approaches.

### 2.1. Direct Experimental Observations of NDs

#### 2.1.1. Model Lipid Membranes

The simplest systems mimicking cellular membrane are lipid bilayers composed of different types of lipids. Understanding on a molecular level the main trends in their structural and dynamic organization under different conditions may shed light on the behavior of real membranes. Studies of mixed lipid bilayers have therefore attracted considerable interest for a long time [19]. The most appropriate system allowing characterization of “microdomains” in the fluid state is a bilayer composed of two phospholipids—under such conditions, many of them demonstrate non-ideal mixing [20,21,22,23]. At the same time, many more experiments have been conducted for more complex systems—lipid bilayers from a mixture of phosphatidylcholines (PC), sphingomyelin (SM), and cholesterol (Chol). This choice is largely due to the fact that this combination of membrane components is characteristic of the formation of well-studied macroscopic high-density lipid domains, such as rafts (e.g., [11] and ref-s therein).

One of the first references to nanoscale molecular clusters can be found in [24]. The term has been used to refer to anomalies observed in a number of physical properties of organic liquids near the freezing point. These clusters were pictured as short-lived dynamic arrangements of adjacent molecules with coordinated movements. It has been proposed that the mean molecular density within the cluster is higher than for freely dispersed molecules, and internal rotational freedom is inhibited for molecules within the cluster. Thus, when a transient cluster is formed, the group of molecules involved moves as a whole during its lifetime, and, for example, the viscosity of the liquid will be greater than that of the hypothetical, cluster-free liquid. The fraction of clusters present in alkanes just above the melting point was estimated as c.a. 10%, with the average cluster size being 3–4 molecules. The presence of clusters has also been used to explain anomalies in the heat capacity data for a number of n-alkanes [25].

The first experimental indications that atomic-scale inhomogeneities are present in model lipid membranes began to be obtained in the 1970s based on the study of model lipid bilayers using X-ray [26], ESR spin-probes [27], and small-angle neutron diffraction [28]. In these works, inhomogeneities in the acyl chain region, including areas bordering the polar heads of lipids were discussed. At the same time, it was formulated that such clusters represent “… short-lived, more densely packed arrangement of molecules within an environment of freely dispersed molecules” [27]. Later it was found (e.g., [29]) that the membrane/water interface also has a similar heterogeneity. This is what will be discussed below.

Important information about transiently stable but mostly dynamic NDs was obtained by a variety of biophysical methods, like Förster resonance energy transfer (FRET) analysis [30,31], interferometric scattering microscopy [32], stimulated emission depletion (STED) microscopy [33], differential scanning calorimetry (DSC) [30], and their combinations. The sizes of the observed ND were in the range of 5–60 nm. In particular, Yano et al. [34] examined the details of their formation by stearoyl-SMs (SSMs) using FRET measurements in lipid bilayers containing SSM and its enantiomer (ent-SSM), dioleoyl-phosphatidylcholine (DOPC), and Chol. In contrast to the uniform domains seen by microscopy, FRET analysis using fluorescent donor- and acceptor-labeled SSM showed distinct differences in SM and ent-SM colocalization within nanoscale distances. It was shown that in the liquid-ordered state (Lo) there exist separate nano-subdomains. The average size of the subdomains decreased as temperature increased, and at physiological temperatures, the subdomains were found to have a 1–10 nm radius and a lifetime of less than 10 µs. deWit et al. [32] succeeded to achieve dynamic imaging of nanoscopic lipid domains without any labels. Using phase-separated droplet interface bilayers they resolved the diffusion of domains as small as 50 nm in radius and observed formation of NDs, destruction, and dynamic coalescence with a domain lifetime of 220 ± 60 ms.

It should be noted that to characterize the dynamic NDs in lipid bilayers, it is necessary that the aforementioned experimental methods operate almost at the limit of their resolution capabilities. Thus, for optical methods, the dimensions of the NDs already lie beyond the diffraction limit. In some cases, an efficient way to increase the resolution consists of combining techniques with different spatial sensitivities. For instance, consistent employment of FRET and small-angle neutron scattering (SANS) permitted to significantly narrow the uncertainty in domain size estimates for DOPC and palmitoyloleoylphosphatidylcholine (POPC) mixtures with SM/Chol [30]. FRET data revealed coexisting domains for both mixtures, while SANS measurements detected no domain formation for SM/POPC/Chol. Together, these results indicate that liquid domains in SM/POPC/Chol are between 2 and 7 nm in radius at 25 °C. Another way to increase the resolution of standard biophysical methods was shown in [35]. The authors applied fluorescence correlation spectroscopy on planar plasmonic antenna arrays with different nanogap sizes to assess the dynamic nanoscale organization of model biological membranes. Taking advantage of the highly enhanced and confined excitation light provided by the nanoantennas, together with their outstanding planarity, they were able to detect membrane regions as small as 10 nm in size with microsecond time resolution. As a result, the measured diffusion data were consistent with the coexistence of transient NDs in both liquid-ordered (L_o_) and liquid-disordered (L_d_) microscopic phases of multicomponent lipid bilayers. These NDs revealed characteristic residence times between 30 and 150 μs and sizes around 10 nm. Coexisting of microscale phase separation with nanoscopic domains led to suggestion that such transient assemblies in model bilayers might be similar to those occurring in living cells, which in the absence of raft-stabilizing proteins are poised to be short-lived.

In contrast to the situation with NDs existing inside larger L_o_ domains, routine experimental methods (e.g., optical spectroscopy) often do not reveal large domains in the water–lipid mixtures. But lateral heterogeneity is nevertheless detected using techniques with nanometer-scale spatial resolution. Enoki et al. [36] proposed a simple and accessible method to measure domain sizes below optical resolution (<200 nm) in distearoylphosphatidylcholine (DSPC)/POPC/Chol and SM/POPC/Chol bilayers via measurements of nanodomain size by combining FRET data with a Monte Carlo (MC)-based analysis. The reported radius of NDs’ was 7.5–10 nm and ~5 nm for the two mixtures, respectively. The existence of NDs within larger L_o_ phase domains has been revealed by fluorescence lifetime and 2H NMR experiments [37]. For example, Raman microscopy observations using diyne-substituted SM have demonstrated that SM-rich subdomains can densely occur in the central area of the Lo domain [38]. The fact that nanometer-sized subdomains often exist inside much larger L_o_ domains makes direct detection of NDs difficult. In order to distinguish the effects of NDs in the experimental data, Pathak et al. [31] proposed to use detergent Triton X-100 in order to induce L_o_ domain formation in SM/POPC/Chol mixtures. Based on the results of nitroxide quenching methods and FRET experiments they showed the existence of NDs in this mixture and their radius gradually decreased from ≥15 to <4 nm as temperature increased from 10 to 45 °C.

Recent achievements in single-molecule technologies made it possible detection of NDs in model membranes with ultra-high spatiotemporal resolution. Thus, Wu et al. investigated organization and single-molecule dynamics of rafts by monitoring lateral diffusion of single molecules in raft-containing reconstituted membranes supported on mica substrates [39]. Using high-speed interferometric scattering (iSCAT) optical microscopy and small gold nanoparticles as labels, motion of single lipids was recorded via single-particle tracking (SPT) with nanometer spatial precision and microsecond temporal resolution. Processes of single molecules partitioning into and escaping from the raft-mimetic Lo domains were directly visualized in a continuous manner with unprecedented clarity. As a result, the presence of NDs in the L_o_ phase was proven in the microsecond timescale. Their characteristic size was about 10 nm. In accordance with the above studies, these results provide direct experimental evidence of non-uniform molecular organization of the Lo phase, giving a new view of how rafts recruit and confine molecules in cell membranes. To overcome the spatial resolution limit inherent in conventional FRET applications, which is determined by the Förster radius (Ro) of the chromophores, Pathak et al. [40] proposed to use a dual-FRET-pair technique in which only one FRET pair had Ro values that were sufficiently small to detect the NDs. Using this approach, they measured the temperature dependence of domain and ultra-nanodomain formation for vesicles composed of various mixtures containing a high-T_m_ lipid (SM) or dipalmitoyl phosphatidylcholine (DPPC)), low-T_m_ lipid (DOPC or POPC) at various concentrations of Chol. (Here, T_m_ is the melting temperature.) The results obtained permitted a suggestion that natural mammalian lipids are tuned to maximize the tendency to form ultra-nanodomains (or NDs) relative to larger domains. The observation that domain size is more sensitive to membrane composition than domain formation has implications for how membrane domain properties may be regulated in vivo.

#### 2.1.2. NDs in Cell Membranes

In contrast to model lipid membranes, which usually consist of several types of lipids and often include Chol or its analogues, there is much less experimental work on the study of NDs in natural cell membranes. First, this is due to the technical difficulties of sample preparation and the study of their domain organization at the limit of the spatiotemporal resolution of the existing modern methods. Second, natural membranes impose strict requirements for instrumental measurements—they must be non-invasive, so as not to affect the viability of cells. In this situation, the most successful to date are optical spectroscopy methods. Thus, Eggeling et al. [41] demonstrated the ability of STED far-field fluorescence nanoscopy to detect single diffusing lipid molecules in nanosized areas in the plasma membrane of living cells. The achieved spatial resolution was ~70-fold below the diffraction barrier. It permitted detection of membrane-anchored proteins, which were transiently (~10–20 ms) trapped in Chol-mediated NDs within <20 nm diameter areas. It was concluded that the non-invasive optical recording of molecular time traces and fluctuation data in tunable nanoscale domains is a powerful new approach to study the dynamics of biomolecules in living cells. Fluorescence burst analysis and fluorescence correlation spectroscopy performed for cell membranes on nanoantennas of different gap sizes gave similar results for SM trapped in Chol-enriched NDs [42] with characteristic size and lifetime—10 nm and 100 μs, respectively.

A biologically relevant and potentially promising approach for in vivo studies of biological nano-objects was recently described in [43]. The authors developed a multimodal label-free imaging platform for measuring intracellular structure and macromolecular dynamics with a resolution ~20 nm/~1 ms. At the same time, being efficient for detection of chromatin structure and dynamics, this method has not yet been tested on cell membranes. The latter are too small to be studied directly with standard biophysical methods, such as optical microscopy. As a consequence, nanoscale characterization of the membranes has been performed ex vivo or in the presence of exogenous labels used to enhance contrast and impart specificity. To overcome this limitation, an isotopic labeling strategy in the gram-positive bacterium Bacillus subtilis has been proposed to investigate the nanoscale structure and organization of its plasma membrane in vivo [44]. The authors labeled the cell and its membrane independently with specific amounts of hydrogen (H) and deuterium (D), which possess different neutron scattering properties without altering the chemical composition of the cells. The cell membranes containing a mixture of H- and D-fatty acids were studied using neutron scattering. By creating neutron contrast within the plane of the membrane, it was possible to detect lateral features smaller than 40 nm.

Although the experimentally-derived information about NDs in vivo is much less detailed than that obtained on model lipid bilayers, the proposed techniques are fully biocompatible and thus provide various new opportunities for biophysics and live cell research to reveal details that in principle cannot be probed in measurements on oversimplified lipid mixtures.

#### 2.1.3. Lessons of Studying NDs in Experiments

Summing up the experimental data on NDs in model and natural membranes, it should be noted that the most reliable methods for detecting nanoscale inhomogeneities are obtained either by the joint application of complementary methods, or by significant modifications of existing approaches with ultra-high resolution. As already noted, the very nature of NDs in dynamic liquid water–lipid mixtures requires the work of experimenters on the verge of possibilities. However, in addition to the emerging technical difficulties, the current situation has a positive side, since it contributes to the rapid development of instrumental technologies, the introduction of new methods of sample preparation, and data analysis, etc.

It is important that, in addition to the already traditional systems containing SM, Chol, and a number of other components that significantly induce the spontaneous formation of NDs (and, therefore, are most convenient for conducting experiments), model membranes made of lipids that are not prone to raft formation are increasingly being studied (reviewed in [11]). Since the mosaicity parameters of the lipid/water interface can vary greatly with changing external conditions, the latter are often modified to achieve the clearest picture. Together with the above-mentioned modifications of the detection methods, this gives the desired result, improving the quality of experimental data on spontaneously formed NDs.

The most important conclusion, which follows from the results of quite numerous independent experimental studies of lateral inhomogeneities in both model lipid bilayers and natural cell membranes, is that NDs actually exist. Their characteristic sizes can be less than 10 nm, and their lifetimes can be up to milliseconds. In addition, experiments have shown that NDs can spontaneously form both in existing larger L_o_ domains and outside of them. Important estimates of their diffusion are also obtained, and in some cases the factors that cause the formation of NDs and affect their size, stability, etc. are determined. This provides a reliable basis for the atomistic analysis of NDs in membranes using computer simulation methods. It is important to note that the first results indicating the spontaneous appearance of nanoclusters in lipid bilayers appeared long before their observation in direct experiments. Apparently, Mouritsen et al. [45] have been the first to predict the formation of metastable nanodomains in DPPC membranes. It is important that the authors concluded that continuum models are insufficient for correct description of NDs and the application of atomistic simulation approaches (in that case, Monte Carlo (MC)) is required. Later, similar results on detection of NDs have been achieved in studies of other single and multi-component PC lipid mixtures of different acyl chain lengths [46,47]. Starting from about 2004, atomistic simulations revealing NDs in different lipid bilayers have become rather common (e.g., [11,48,49]).

Therefore, for a long period of time, such computational work on the properties of NDs aroused natural skepticism, given the number and nature of approximations originally inherent in the in silico methods. Therefore, right now, in connection with the experimental confirmation of the ND-related effects, there is a great opportunity not only to generalize the accumulated calculated data and correlate them with those observed in experiments, but also to start joint coordinated research on the topic under consideration. Such work has already begun (see below). An excellent presentation of the computational work already carried out on the analysis of NDs in membranes is given in the reviews [11,50,51]. Therefore, here we do not repeat in detail what was said earlier, but focus on issues that have been relatively less described in the literature. Namely, we discuss the problems of clustering in the identification of NDs and the description of their dynamics, as well as mapping and visualization of the mosaic picture of the membrane surface. Finally, it is of great interest to analyze the changes in the DMP depending on the composition of the membranes and other factors.

### 2.2. Computer Simulations

Based on the results obtained using computational methods, the following conclusions can be formulated regarding the conditions of existence and spatiotemporal characteristics of NDs in model membranes:

(1) Computer simulations of model membranes clearly indicate the existence of NDs on the lipid bilayer surface. Moreover, such objects were first discovered “at the tip of the pen” (i.e., in silico), and only later observed in the direct experiments described above and others. The characteristic sizes and lifetimes of the calculated NDs are >1 nm and >1 ns, respectively, which is perfectly consistent with the results of observations. It is important that the computational data about the mechanisms of NDs formation and the influence of various factors on them (environmental conditions, etc.) are reproduced for the same systems using different calculation technologies: force fields, the level of approximations used (all-atom, united-atom, coarse-grained, etc.), computational protocols of sampling, and other modeling parameters. This indicates that the NDs are not an artifact caused by the choice of computational methods for obtaining data and processing them;
(2) In computer models, it is possible to reproduce well the effect on the DMP of model lipid bilayers of such factors as temperature, the chemical nature of lipids in the membrane, a certain ionic composition, the role of the opposite monolayer, the presence of embedded “alien” objects with different parameters of mobility, etc.

Thus, there is every reason to expect that modern modeling methods can now be used to predict such properties of NDs that cannot yet be investigated in direct experiments. Once again, we note that this is a fundamental point, since before a clear experimental confirmation of the phenomena under consideration, any in silico attempts to improve the liquid–mosaic model of membranes by aligning it with the concept of NDs were only hypothetical and, therefore, caused healthy skepticism. Now, the use of computer modeling allows us to study the following ND-mediated phenomena and make predictions that can be further tested experimentally:

- Analyze in detail all types of interactions in the membrane, both at the level of individual molecules and the entire ensemble, and evaluate their contribution to the DMP characteristics. This allows determination of the balance of forces that cause the formation of the NDs with the observed properties in each particular case;
- The possibility of pictorial visualization of the DMPs. They can be presented both in the form of beautiful 3D images, and in the form of much more informative 2D maps of the membrane surface (e.g., [52,53]). In both cases, animation is widely used to represent the dynamics of these complex objects. It is important that the membrane system can be depicted as a whole, or the emphasis can be placed on the behavior of its individual components, for example, NDs, free volume regions, single molecules/groups. In addition, using numerical mapping methods, it is possible to graphically represent a wide variety of physico-chemical characteristics associated with NDs. These include: hydrophobic/hydrophilic and electrical properties (for example, molecular hydrophobicity potential (MHP) or electrostatic potential (EP), charge density, etc.); surface topography and its degree of hydration; mobility of molecules and their individual groups (in particular, calculated during MD); characteristics of various types of interactions involving membrane components (for example, the density of H-bonds, salt bridges, etc.). On such maps, it is useful to plot information about the location of specific atoms and molecules, the area of contact with external agents—for example, binding peptides, proteins, and so on. It is important that the presented surface properties can relate to individual states of the system, as well as to their average values, difference values, etc.;
- Calculate with ultra-high spatiotemporal resolution the motion characteristics of all components of the membrane, up to individual atoms and groups, identify collective movements in the bilayer, quantify their contribution to the integral picture, and predict the influence of specified external factors on them;
- In addition to the lateral NDs, to study such inhomogeneities along the normal to the membrane plane (the first attempts have already been made [54]). Together, the information obtained allows for the creation of an unprecedented detailed 3D dynamic picture of model cell membranes on a nanoscale;
- Consider a wide range of systems in the calculations—from model hydrated bilayers consisting of one or more types of lipids to native-like membranes. Of course, the vast majority of results have so far been obtained for the first case, although it is not so much the less computational complexity of modeling such systems (this is, obviously, an important factor, but it is not critical). The key point is the need to constantly calibrate the results of calculations based on experimental data, and the latter are now available only for such simple systems. In addition, since detailed physical mechanisms for the formation and existence of domains of different scales (including NDs) have not yet been established, modeling of relatively simple systems allows much better control of the contribution of individual factors (for example, lipid composition, the presence of ions, etc.) to the observed phenomena. For the most complex multicomponent membranes, such an analysis is still impossible. The reason is the lack of reliable experimental structural data and other properties for them, on the basis of which it would be possible to carry out parameterization and verification of force fields and simulation protocols.

From a methodological point of view, the rich experience of applying computer modeling methods in the study of nanoscale-resolution membranes can be summarized as follows:

- The most commonly used is the molecular dynamics (MD) method in its different implementations: classical and Langevin MD, targeted MD, steered MD, and so on. The Monte Carlo (MC) method is still used, although to a lesser extent than before. In addition, there are known examples of the joint application of the MD and MC methods. In relation to the problems of studying membrane DMPs, MD methods seem to be the best choice, since the dynamic aspects of NDs are extremely important —without taking them into account, it is impossible to identify the mechanisms of the phenomenon and make its complete atomistic picture (see below). The availability of several well-developed and constantly supported modeling programs (GROMACS [55], NAMD [56], CHARMM [57], etc.) and the open-source practice used in their implementation also played a major role in the popularity of MD, which allows users to actively implement their developments within these software products;
- Adequate and consistent with both the experiment and the independent modeling studies, the results are obtained using at least several modern force fields: GROMOS96 (with “Berger lipids”) [58], CHARMM36 [59], SLipids [60], MARTINI [61], etc. It is important that these energy functions were specially adapted to the calculations of systems containing all the main components of the cell membrane—lipids, water, proteins, sugars, as well as physiologically significant ions. This allowed us (at least in the main) to get rid of the previously common problems of inconsistent joint application of force field parameters, for example, for lipids and proteins. In contrast to problems that consider only the integral parameters of the membrane, and, therefore, in many cases, are not too sensitive to such details, this question plays an extremely important role (sometimes critical) when it comes to analyzing the interactions of individual molecules, which leads to the formation/decay of NDs, their diffusion, etc.;
- Speaking of force fields, it should also be noted that in the analysis of NDs, different levels of approximations are used: all-/united-atom and coarse-grained (CG) models, as well as, of course, their combinations. In contrast to “conventional” (i.e., non-nanoscale analysis) MD calculations, continuum models are much rarely used now, since individual molecules (water, lipids, and ions) play a key role in the processes under study. In some cases, modeling of complex systems (for example, a protein embedded in a membrane) begins with the use of continuous models of the membrane to find the starting states for further calculations in an explicit solvent;
- Depending on the level of approximations, the duration of MD trajectories, which can currently be achieved on available high-performance clusters (including personal computers with additional GPU cards), reaches tens of microseconds for all-atom models and up to millisecond scale for CG models. In this case, as a rule, several independent MD runs are required for the same system.

A joint analysis of the entire set of experimental and in silico results of detection of NDs spontaneously formed in model lipid bilayers and in native-like membranes indicates a good agreement of the observed pattern of lateral heterogeneity of the membrane-water interface. This applies to the size of the NDs, their lifetimes, composition, and other characteristics. What factors determine this picture? This is discussed below.

## 3. Molecular Mechanisms of NDs Formation

### 3.1. Free Energy of Lipid–Lipid Interactions

In binary mixtures, spatial distribution of lipids in the plane of the membrane and formation of ideal/nonideal solution is determined by the unlike nearest-neighbor interaction free energies (ω_AB_) between lipids in thermodynamic equilibrium [19,62]. Interactions between two types of lipids may be repulsive (ω_AB_ > 0), which means that lipids are likely to make contacts with the similar ones. Strongly repulsive interactions result in formation of domains. If interactions are slightly repulsive, there exist micro-heterogeneities in the system, which do not grow to domains. Ideal solution occurs only in case the lipids do not have any preferences in the nearest neighbor lipid types (ω_AB_ = 0). Lipids mix even more uniformly than in random mixture if they prefer nearest neighbors unlike themselves (ω_AB_ < 0). Experimentally, the values of ω_AB_ can be estimated, e.g., with the help of isothermal titration calorimetry, differential scanning calorimetry, NMR, and FRET. Such data for a number of binary lipid–lipid and lipid–cholesterol mixtures are given in [19]. Apart from the values of ω_AB_, experiments can show whether NDs are composed of similar or different types of lipids, thus forming homo- or heterophilic clusters. For example, a confocal microscopy study of the mixtures SM/ent-SM/Chol [63] demonstrated that homophilic SM interactions, because of the stereospecific intermolecular attraction, induce the formation of nano-subdomains in the L_o_ phase of SM-based bilayers. In addition, it was found that this effect is mediated by Chol, which has a strong ordering effect on SMs [64] and formation of NDs [63]. However, such an information about the type of the nearest interaction neighbors is integral—it does not provide reliable indication of the physical origin of these phenomena.

Hence, analysis of inter-molecular contacts between all components of the membrane (lipids, water, ions, and so on) is required. These include mainly H-bonds, van der Waals and ionic interactions. The former were shown to play especially important role in formation of nanosized clusters (or NDs). This topic is considered in the next section.

### 3.2. Role of H-Bonds in Formation of NDs

It is well established now that the free energy of the H-bond strongly depends on the local dielectric properties of the medium (e.g., [65]). Thus, the strength of the H-bond increases when the interacting groups are immersed in the nonpolar region of the membrane, where the values of the dielectric constant (ε) are much lower than in water [66]. The most significant contribution to the interlipid H-bonds is made by the NH-groups of SM and the carbonyl groups of phospholipids [67,68]. This is because, among all H-bond donors/acceptors, these groups are located in the most nonpolar environment in the bilayer. In addition, the interactions of phosphate groups also play an important role. Even though many lipids (for example, PCs) do not have H-bond donors, strong interactions between them are carried out by water molecules and ions (e.g., [69]).

From the point of view of ND formation, both the spatial location of H-bond donors/acceptors in neighboring lipids, the possibility of their hydration, and the distribution of charged groups (the latter are very important for ion binding) are of great importance. Thus, the final picture of the location of the polar heads of lipid molecules in the membrane plane is determined by the balances of a large number of intermolecular interactions that contribute with different weights. However, in addition to direct contacts at the membrane–water interface, it is necessary to take into account the effect of the acyl chains of lipids on the surface mosaicity. It is shown that the nature of their packing and movement can influence the NDs. Thus, the lateral heterogeneity on the nanoscale is characteristic not only of the region of the polar heads of lipids, but also of the inner nonpolar regions of the membrane [54].

**Figure 1:**
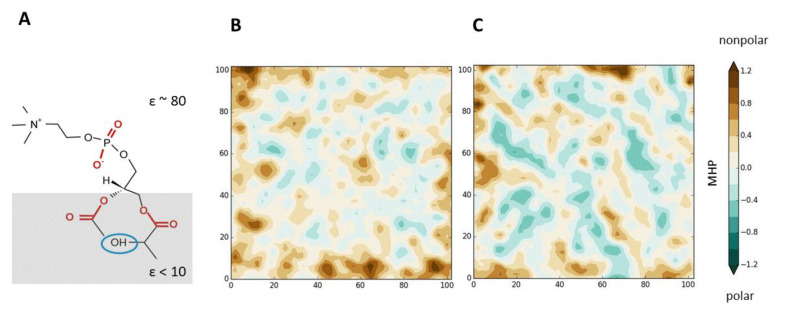
(**A**) Schematic representation of location of sn-1-β-hydroxy-dioleoylphosphatidylcholine molecule (DOPC-oh) with respect to the membrane. The low-dielectric part of the membrane is gray hatched. Only polar head of DOPC-oh is shown. Engineered H-bond donor is blue circled. H-bond acceptors are given in red. ε is the dielectric permittivity. (**B,C**) Hydrophobic/hydrophilic organization of the surfaces of DOPC (**B**) and DOPC-oh (**C**) bilayers (according to [49,74]). In-plane projections of the distributions of the molecular hydrophobicity potential (MHP) values over the solvent accessible surface of lipid bilayers (2D-maps of MHP). MHP values are calculated in each point of the surface. The maps are given for one of the bilayer leaflets. Bilayer snapshots are extracted form MD trajectories of the corresponding bilayers. Coloring scheme for MHP is shown on the right. MHP values are given in octanol-water log *p* units.

Recently, important results have been obtained on the parameters of the H-bonds of lipids included and not included in the NDs. In addition, some factors affecting the patterns of H-bonds within the NDs and at their boundaries were identified. In addition to the chemical nature of the donor/acceptor groups and the degree of polarity of their local environment (i.e., ε), both in these lipids themselves and in their neighbors, the structural and dynamic characteristics of these lipids, their ability to form sterically tight contacts, play an important role in the analysis of the lipid molecules forming NDs. Often, a kind of “stimulator” of the formation of NDs is Chol. In particular, Yano et al. showed [34] that in mixtures with SM, Chol enhances the formation of H-bonds between SM molecules, limiting the conformational mobility of their acyl chains in the nonpolar region of the bilayer [70,71]. In turn, this contributes to the formation of NDs inside the L_o_ phase of the membrane. Interestingly, the Chol molecules themselves may not be part of the ND. Similar effects of inducing the clustering of certain types of lipids by their neighbors in the bilayer determine the features of the molecular composition of NDs. Often, they mainly involve lipids of the same type (homo-clusters) [63], or, conversely, lipids of different types (hetero-clusters) [72]. To obtain the above information, both experimental methods (for example, FRET with specially selected chromophore groups) and computational approaches are used. In both cases, a powerful tool for deciphering the nature of intermolecular interactions is the use of artificial lipids with selective modifications that affect the composition of H-bond donors/acceptors. Thus, it was shown that N- and O-methylation of sphingomyelin markedly affects its membrane properties and interactions with cholesterol. In particular, such a blockade of H-bond donor and acceptor groups in SM disrupts ordered domain formation [73]. This indicates the importance of the amide group of SM as a primary force in SM-specific appearance of NDs. This idea was further developed in our design of an artificial DOPC-based lipid with an altered pattern of H-bonding, which could potentially affect properties of a pure DOPC bilayer. The aim was to introduce an H-bond donor (OH-group) into the DOPC molecule at a depth of C = O groups of an unmodified lipid (Figure 1A). The resulting lipid (sn-1-β-hydroxy-dioleoyl phosphatidylcholine) was further designated as DOPC-oh. According to our estimates [68] based on MC calculations in media of various polarity (with different ε), it is in the region of the location of the C = O groups of DOPC—i.e., in a nonpolar environment (ε ~3)—that the strongest effect of the formation of additional H-bonds with the participation of DOPC-oh molecules is observed. MD simulations of lipid bilayers containing DOPC-oh have demonstrated that the addition of this compound to the DOPC membrane leads to significant changes in a number of the most important structural and dynamic properties of the bilayer that determine its DMP: a strong increase in the “contrast” of the lateral distribution of the hydrophobic/hydrophilic properties of the membrane/water interface, a change in its domain structure (parameters of NDs), H-bond patterns, and mobility (in preparation). It is important to note that such “reporter” molecules of artificial lipids can significantly change the macroscopic average properties of the bilayer surface. This is clearly seen in Figure 1B,C, which shows the averaged maps of the distribution of hydrophobic/hydrophilic properties (expressed in terms of MHP) on the surface of the DOPC bilayer and the bilayer containing DOPC-oh. Such effects are described for the first time, and this opens up new possibilities in the rational design of artificial membranes with specified properties.

### 3.3. Stochastic Fluctuations

An interesting and important feature of NDs in membranes is the difference in their behavior from large-size heterogeneities, including rafts. Thus, an increase in temperature leads to the suppression of the effects of macroscopic separation of the L_d_ and L_o_ phases in the lipid bilayer due to more efficient mixing of lipids. At the same time, the formation of short-lived nanosized structures (i.e., NDs) is enhanced [75]. These phenomena are particularly pronounced near the phase transition temperature (Tm) of the lipids involved. Indeed, such phenomena have been observed for both giant plasma membrane-derived vesicles [76] and synthetic liposomes [77], further supporting the use of model systems to understand the heterogeneity in the plasma membrane. It is generally assumed that the main role in these processes is played by the so-called “critical fluctuations”—stochastic thermal movements of the bilayer components (lipids, water, ions, etc.), leading to the appearance of free volume zones, collective nanoscale movements, abnormal diffusion of lipids and water, etc. It is shown that artificially created immobilization of individual components of the bilayer (for example, the introduction of limited mobility of lipid molecules [78]) can significantly “quench” such fluctuations, which ultimately leads to the suppression of the processes of formation of NDs. This is discussed in the next section.

### 3.4. Effects of Linactants

The above-described method of affecting the distribution of NDs on the lipid bilayer surface by introducing molecules with a changed pattern of H-bond donors/acceptors is not the only one. There are other reasons why an “alien” object inserted into the membrane (for example, an artificially created molecule, including hybrids of different lipids) promotes lateral clustering on a nanoscale. Thus, the nature of the acyl chains of such added lipids plays an important role: “hybrid” lipids with one unsaturated and one saturated chain might promote nanodomain formation, and they can partition to phase boundaries and act as a linactant. Linactant is a 2D analogy to surfactant and is hence a molecule that reduces the line tension of an interface, thus disfavoring the coalescence of small domains into larger ones and all the way to microscopic phases. Hybrid lipids reduce the line tension of phase boundaries by partitioning efficiently into both phases and reducing their structural dissimilarity [79]. However, this picture has also been challenged by experiments [80]. Finally, the effects of ND stimulation by molecules that differ in terms of mobility and/or hydrophobicity from bilayer molecules are known.

Thus, immobile objects, such as “frozen” lipids or pickets (e.g., transmembrane protein segments), seem to strongly destabilize phase separation, while freely diffusing membrane-spanning objects such as peptides can promote phase separation via the hydrophobic matching mechanism. Regarding the former case, Fischer et al. simulated the dilineoylphosphatidylcholine (DLiPC)/DPPC/Chol mixture in the coarse-grained scheme and examined the role of immobile obstacles on phase separation [78]. It was shown that restraining of DPPC lipids either in the plane of the membrane in both leaflets or in all dimensions but only in one leaflet allowed or suppressed undulations perpendicular to the membrane plane, respectively. Immobilized lipids prevented full phase separation, which was otherwise observed. The second phenomenon (addition of pickets) highlights the importance of considering the effects of proteins on membrane phase behavior. Because here we consider only spontaneously formed NDs, this topic lies beyond the scope of the present review.

### 3.5. Diffusion, Collective Moves, and Free Volume Zones in Membranes

In addition to the spatiotemporal parameters of the NDs, the diffusion of the bilayer components is of great importance when considering the factors that determine the DMP (mosaicity) of the membrane surface. The problem becomes particularly relevant in view of the fact that at the scale under consideration, the nature of the motion of the bilayer components differs from the Brownian motion observed for macroscopic systems. Anomalous diffusion effects of lipids, ions, and water occur. In addition, nanoscale collective movements were registered, etc. [81,82,83]. For more details, see the reviews [10,13]. These parameters of the local diffusion of lipids, water, and ions in the surface layer and in the system as a whole are directly related to the spontaneous formation of NDs. Thus, it has long been shown [11,13,84] that at the nanoscale, i.e., at the level of individual molecules and groups of several lipid molecules, lateral diffusion in model membranes (there is simply no data on real ones yet) has a non-Brownian character. On the surface of the membranes, micro-flows, vortices, etc. are constantly forming and disappearing. The characteristic lifetimes of such phenomena are from 1 ns and higher. Undoubtedly, the formation of NDs and such collective movements are due to a common physical mechanism, the details of which are still not fully understood. At the same time, it is shown [81,82] that in some cases, lipids inside NDs move consistently, but more slowly than non-clustered bilayer molecules. It is well known (see, for example, the review [11] and ref-s therein) that the processes of diffusion in membranes and, consequently, the pattern of lateral clustering on the nanoscale are largely determined by the free volume zones in the lipid bilayer that arise due to fluctuations. In particular, it was found that they determine the abnormally fast diffusion of single water molecules in the membrane, and the degree of hydration of individual lipid molecules, which leads to the formation of NDs. Note that these effects are usually studied using atomistic modeling, since it is extremely important to observe the behavior of single molecules and their small groups (for example, from 3–5 lipids).

### 3.6. NDs and Interdigitation of Acyl Chains of Opposite Lipid Monolayers

How much does the ND pattern in one of the monolayers of the lipid membrane depend on the second monolayer? One of the reasons that allows answering this question in the affirmative is purely mechanical. The fact is that in model lipid membranes, mutual penetration of acyl chains of lipids of opposite monolayers into each other (so-called interdigitation) is often observed, both in experiments and in calculations. However, its role in the behavior of NDs is poorly understood. There are no direct experimental data on this subject yet, but some conclusions can be made on the basis of modeling. Thus, using CG MD, it was shown that the DMPs of monolayers correlate (at least partially) with each other [51]. For example, simulations have demonstrated that the formation of a domain in one leaflet can induce heterogeneity in the other leaflet that does not phase separate by itself. In addition, when fluctuations were present, multiple domains formed in the membrane, and they did not coalesce. Suppressing the fluctuations and inducing immobilized lipids only in one leaflet resulted in intermediate behavior between macroscopic phase separation and nanodomain formation. These effects are discussed in more detail in the review [11].

For natural membranes of living cells, such data are simply not yet available. With the exception of the first successful attempts to register NDs in such systems and estimate their size/lifetime, detailed data on the atomic scale are still not available, and conclusions about the role of the factors discussed above cannot be drawn due to the extreme complexity of such membranes.

## 4. Correspondence between Experimental and Computational Data

As indicated above, both experimental and computational methods contribute significantly to our understanding of the physical nature of lateral NDs, their dynamics, and biological impact. The joint use of these approaches makes it possible to obtain the most adequate information about NDs from independent sources. In addition, such self-consistent analysis contributes to the mutual enrichment of computer and “wet” experiments, thus ensuring rapid progress in this field. At the same time, bridging simulation and experiment is not always a simple task (see [51] for recent review).

Let us highlight the most significant problems: (1) It is very difficult to apply exactly the same conditions for observing NDs in both approaches. This is necessary in order to compare the results of calculations with experimental data and make mutual adjustments in case of discrepancies. We are talking, in particular, about the molecular/ionic composition of the medium surrounding the lipid bilayer, and the observation times (in all-atom MD, they are still limited to microseconds). In addition, the calculations have difficulties with the parametrization of force fields for molecules used in the experiment, but for which there is no reliable structural data to create, for example, the topologies necessary for calculating the energy. The correct accounting of the ionization states of the molecules, including the local pH values, is a particular difficulty. Under experimental conditions, only the integral values of these parameters can be controlled (including the data obtained using molecular pH-sensors), although the charge states of individual components of the membrane can change. (2) As mentioned above, special reporter groups—probes of various nature (fluorescent, spin, etc.)—are often used in experimental studies of NDs, which can distort the finely regulated interactions in the membrane. (3) The quality of the conformational sampling in the calculations of the most complex membrane systems containing up to 10^8^ particles does not guarantee the accumulation of a representative ensemble of states—with all the ensuing consequences. This is also due to the limited size of the objects available for analysis, which in many cases does not allow the calculations to correctly reproduce a number of fundamental properties of the membranes, such as the shape of the entire surface and the roughness of different scales that occur on it (the so-called curvature effects). (4) Both experimental and computational approaches have their own errors and limitations, some of which are extremely difficult (sometimes impossible) to control, which therefore makes it questionable direct comparison of the results. (5) Although today we are struggling to understand at the molecular level the structure of simple model membranes, modern experimental technologies go much further, allowing in vivo studies of the biological aspects of the functioning of more and more complex membrane systems, where the most interesting phenomena occur in the cell. This creates a significant gap between the real needs of biomedicine and the capabilities of rigorous physical methods, which are so far effective only with simple models. These include all the computer approaches used today. Therefore, many of the results of experiments on the membranes of living cells cannot yet be reproduced in the simulation.

## 5. Representation of NDs via Mapping the Membrane Surface

When analyzing DMPs, it is important to correctly and clearly display information about the time-dependent transient nature of the NDs and other areas at the membrane–water interface. There are various formats for describing NDs—they can be represented both as density clumps and as sections of the lipid bilayer surface with particular physico-chemical properties. In the first case, the clustering of atoms and/or molecules in the membrane is performed according to a geometric criterion, i.e., taking into account the distances between the participants of the NDs. It is noteworthy that this procedure is not trivial—the choice of numerical criteria of the clustering algorithm plays an important role. In particular, clusters can be detected at the level of full-size lipid molecules (e.g., [72]), but the picture of NDs may be distorted due to the conformational flexibility of lipids, which also varies greatly along the normal to the membrane plane. Thus, in the interfacial region, as a rule, strong interactions of the polar lipid heads with each other and with water and ions are observed. As a result, the mobility in this bilayer part is significantly less than in the acyl chain region. Consequently, NDs defined according to the geometric criterion and including the entire lipid molecules may be too heterogeneous in their packing: they have areas of high density at the interface and rarefaction in the acyl chain zone [54]. Therefore, in many cases, it is more correct to perform clustering taking into account not the entire molecules, but only their individual atoms/groups that meet the distance criterion, regardless of which lipid molecules they belong to. This gives a physically more relevant picture—the spatiotemporal distribution of the density of a particular type of atom in the membrane. Finally, in addition to high-density regions, important information is provided by localization and visualization of low-density regions—the so-called free volume zones, which play a key role in the processes of diffusion of membrane components, protein–membrane and protein–protein interactions in the lipid bilayer. Another difficulty in describing NDs is related to the quantitative characterization of their lifetimes. The fact is that in the course of its existence (registered, in particular, in the MD), the composition of the ND changes gradually—some members of the group leave it, others appear. The question arises as to how exactly to estimate the lifetime of such dynamic groups. To solve the problem, cutoffs are used for the time of the presence of individual specific components in the ND: for example, if one is present in the cluster for more than 90% of the observation time, then it is considered a member of the group. In addition, the concept of “core” and “periphery” of the cluster is used, introducing different cutoff values for them.

**Figure 2:**
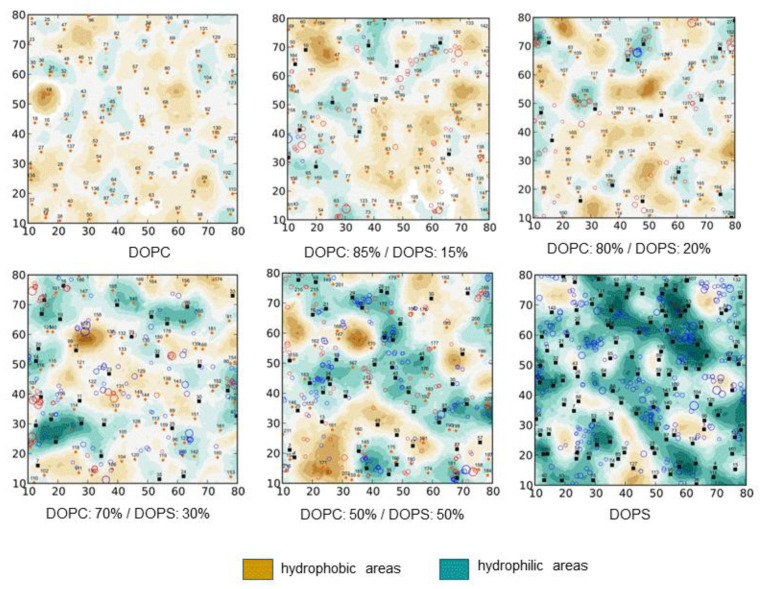
Dependence of the “dynamic molecular portrait” (DMP) of the DOPC/DOPS lipid bilayers on the DOPC:DOPS ratio. Results of all-atom MD simulations. DMPs are given in terms of 2D-maps of MHP of the bilayer surface (see legend to Figure 1) averaged over MD trajectory. Bilayer content is indicated under each panel. In-plane projections of lipid centers of mass are shown with small circles. Other MD details are described in [72].

In the second approach, the distribution of properties (for example, MHP, EP, landscape, mobility, the number of H-bonds of a certain type, etc.) is calculated at the points of the surface, and then the resulting picture is analyzed, identifying groups of surface points with similar characteristics. In this case, clustering by properties can be performed both by working with the 3D distribution of surface points, and initially projecting the property parameters on a plane parallel to the plane of the membrane surface. The transition from a 3D picture to a 2D representation—to the so-called maps of the physico-chemical properties of the membrane surface—provides a number of useful opportunities for the analysis of DMPs. First, such maps can be presented in the same format for different systems (for example, lipid bilayers of different composition, Figure 2), and for a set of states of the same system, but under different conditions (for example, during MD). This makes it easy to compare maps with each other—calculate difference maps, perform averaging of the maps and calculate standard deviations, etc. Secondly, in addition to the ND-mediated characteristics themselves, auxiliary information can be mapped, for example, marking different types of molecules, areas of a particular degree of hydration, and so on.

## 6. Conclusions

Concluding the story about NDs and related phenomena of spontaneous lateral clustering in lipid bilayers, it should be noted that the presented DMPs are specific for each membrane system of a given composition and considered under particular conditions (temperature, pressure, degree of hydration, etc.). Visual and informative tools of mapping and visualizing the surface properties of model lipid bilayers allow quick and reliable drawing of conclusions about both the integral characteristics of the membrane (for example, the blurred or, conversely, the contrast distribution of MHP or EP), and about the nanoscale parameters, expressed, e.g., in the distributions of NDs by size, lifetime, chemical composition, types of intra-and intermolecular interactions, etc. (e.g., [53]). The uniqueness of the DMPs allows us to not only rationally explain the differences in the properties of lipid bilayers (which in itself is of fundamental importance), but also to design new membrane materials with specified properties, in particular, by varying their composition and/or external conditions.

The above-mentioned feature of the model lipid bilayers to vary the parameters of the DMPs (expressed in terms of the lateral distribution of NDs), apparently, is one of the most important fundamental properties of the membranes, since it allows them to very smoothly adjust their surface properties, reacting to external conditions. If in the case of spontaneous NDs, we are considering, we are talking about such factors as temperature, lipid composition, ion profile, etc., then a number of additional factors appear in natural membranes that strongly affect the picture of DMPs. Among them, of course, the main role is played by integral proteins already present in the membrane, peptides, proteins and other molecules binding on the surface, etc. It is known that in such processes, local areas of the membrane (primarily those in contact with such external agents) experience significant disturbances, adapting to “alien” agents (e.g., [85,86]). The mechanisms of these processes are directly related to the formation/rearrangement of the NDs pattern. However, this is the subject of a separate consideration. Some important aspects of the biological impact of ND-related phenomena are discussed in excellent recent reviews [10,11,13,51].

## Abbreviations

2D	Two-dimensional
3D	Three-dimensional
CG	Coarse-grained
Chol	Cholesterol
DLiPC	Dilineoylphosphatidylcholine
DMP	Dynamic molecular portrait
DOPC	Dioleoylphosphatidylcholine
DOPC-oh	sn-1-β-hydroxy-dioleoylphosphatidylcholine
DOPS	Dioleoylphosphatidylserine
DPPC	Dipalmitoylphosphatidylcholine
DSPC	Distearoylphosphatidylcholine
ent-SSM	Enantiomer of stearoyl-sphingomyelin
EP	Electrostatic potential
ESR	Electron spin resonance
FRET	Förster resonance energy transfer
L_d_	Liquid-disordered phase in lipid bilayer
L_o_	Liquid-ordered phase in lipid bilayer
MC	Monte Carlo method
MD	Molecular dynamics
MHP	Molecular hydrophobicity potential
ND	Nanodomain
NMR	Nuclear magnetic resonance
PC	Phosphatidylcholine
POPC	Palmitoyloleoylphosphatidylcholine
SM	Sphingomyelin
SSM	Stearoyl-sphingomyelin
